# Public perception of psychiatry, psychology and mental health professionals: a 15-year analysis

**DOI:** 10.3389/fpsyt.2024.1369579

**Published:** 2024-04-30

**Authors:** Javier Domingo-Espiñeira, Andrea Varaona, María Montero, Francisco J. Lara-Abelenda, Luis Gutierrez-Rojas, Elena Ameyugo Fernández del Campo, Roberto Rodriguez-Jimenez, Mariana Pinto da Costa, Miguel A. Ortega, M. Alvarez-Mon, Miguel Angel Alvarez-Mon

**Affiliations:** ^1^ Department of Medicine and Medical Specialities, University of Alcala, Alcala de Henares, Spain; ^2^ Departamento Teoria de la Señal y Comunicaciones y Sistemas Telemáticos y Computación, Escuela Tecnica Superior de Ingenieria de Telecomunicación, Universidad Rey Juan Carlos, Fuenlabrada, Spain; ^3^ Psychiatry Service, Hospital Universitario San Cecilio, Granada, Spain; ^4^ Department of Psychiatry and CTS-549 Research Group, Institute of Neurosciences, University of Granada, Granada, Spain; ^5^ Sleep Unit, Clinical Neurophysiology Service, San Carlos University Hospital, University Complutense of Madrid, Madrid, Spain; ^6^ CIBERSAM-ISCIII (Biomedical Research Networking Centre in Mental Health), Madrid, Spain; ^7^ Department of Psychiatry, Instituto de Investigacion Sanitaria Hospital 12 de Octubre (imas12), Madrid, Spain; ^8^ Department of Legal Medicine and Psychiatry, Universidad Complutense de Madrid (UCM), Madrid, Spain; ^9^ South London and Maudsley NHS Foundation Trust, London, United Kingdom; ^10^ Institute of Psychiatry, Psychology & Neuroscience, King's College London, London, United Kingdom; ^11^ Ramón y Cajal Institute of Sanitary Research, Madrid, Spain; ^12^ Immune System Diseases-Rheumatology and Internal Medicine Service, University Hospital Príncipe de Asturias, Centro de Investigación Biomédica en Red, Enfermedades Hepáticas y Digestivas (CIBEREHD), Alcalá de Henares, Spain; ^13^ Department of Psychiatry and Mental Health, Hospital Universitario Infanta Leonor, Madrid, Spain

**Keywords:** mental health discourse, psychiatry, psychology, psychiatrist, psychologist, social media, analysis

## Abstract

**Background:**

X (previously known as “Twitter”) serves as a platform for open discussions on mental health, providing an avenue for scrutinizing public perspectives regarding psychiatry, psychology and their associated professionals.

**Objective:**

To analyze the conversations happening on X about psychiatrists, psychologists, and their respective disciplines to understand how the public perception of these professionals and specialties has evolved over the last 15 years.

**Methods:**

We collected and analyzed all tweets posted in English or Spanish between 2007 and 2023 referring to psychiatry, psychology, neurology, mental health, psychiatrist, psychologist, or neurologist using advance topic modelling and sentiment analysis.

**Results:**

A total of 403,767 tweets were analyzed, 155,217 (38%) were in English and 248,550 (62%) in Spanish. Tweets about mental health and mental health professionals and disciplines showed a consistent volume between 2011 and 2016, followed by a gradual increase from 2016 through 2022. The proportion of tweets discussing mental health doubled from 2016 to 2022, increasing from 20% to 67% in Spanish and from 15% to 45% in English. Several differences were observed on the volume of tweets overtime depending on the language they were written. Users associated each term with varied topics, such as seeking for help and recommendation for therapy, self-help resources, medication and side effects, suicide prevention, mental health in times of crisis, among others. The number of tweets mentioning these topics increased by 5-10% from 2016 to 2022, indicating a growing interest among the population. Emotional analysis showed most of the topics were associated with fear and anger.

**Conclusion:**

The increasing trend in discussions about mental health and the related professionals and disciplines over time may signify an elevated collective awareness of mental health. Gaining insights into the topics around these matters and user’s corresponding emotions towards them presents an opportunity to combat the stigma surrounding mental health more effectively.

## Introduction

Mental health has been shown to be encumbered by stigma and misconceptions in many past studies (1–3). Prevalence of mental disorders has only increased throughout the years, constituting a major global problem and the leading cause of disability worldwide (4–11). Addressing stigma around mental health is imperative to enhance therapeutic adherence, alleviate symptoms, and promote effective treatment.

In recent decades, social media has served as a tool for identifying and better understanding the population’s concerns about mental health, as it provides a space where people share opinions and engage in discussions on health topics (12–15). Its real-time and unfiltered nature due to its anonymity provides an ideal medium for individuals to express their sincere thoughts and emotions, making X a potential and non-intrusive source of information for researchers (13, 16, 17).

Recent studies have concentrated on examining the global concept of “mental health” or “mental illness” on X (previously known as "Twitter") through the analysis of English tweets exclusively (2, 18, 19), omitting discussions on mental health conveyed by users in other languages. From these studies, one has centered on analyzing tweets posted during the promotion of a mental health awareness campaign conducted for a week in 2017 while other focuses on analyzing tweets for one year (2, 18). On the other hand, other investigations, despite spanning a decade, do not encompass tweets beyond 2017 (19). While these investigations acknowledge discussions surrounding “mental health” as a global concept, one aspect is that they do not analyze more specific constructs within mental health discourse, such as conversations about mental health professionals (psychologists and psychiatrists) or disciplines (psychology and psychiatry) on a specific level. Furthermore, analyzing tweets in other languages would widen the gap in understanding if opinions and discussions about mental health, professionals, and disciplines differ across various language-speaking users over an extended period, updating information over the last five years.

Analyzing public discourse on X concerning psychologists, psychiatrists, psychology, and psychiatry plays a pivotal role in shaping mental health practice, policy, and awareness. Identifying public misconceptions and concerns about mental health professionals and disciplines provides invaluable insights into societal attitudes, essential for developing more informed interventions that resonate with public sentiment. Elaborating interventions aimed at demystifying mental health professionals and disciplines and fostering empathy can encourage timely help-seeking behavior and open engagement with mental health services, facilitating early diagnosis and more effective treatment outcomes.

Our study focuses on the evolving discourse surrounding mental health and mental health professionals and disciplines, aiming to understand the dynamics of public opinion and emotions as expressed on X. By analyzing tweets in English and Spanish over an extensive timespan, we seek to discern shifts in public perceptions, identify recurring topics of discussion, and illuminate the sentiments associated with these topics.

## Materials and methods

### Study design, search strategy and data collection on X

In this retrospective study, we selected X as our database as it is predominantly text-based, facilitating the analysis of written content. Additionally, X serves as a leading platform for diverse discussions on a variety of topics, offering publicly accessible data conducive to our research needs. We collected and analyzed all tweets posted in English or Spanish between 2007 and 2023 referring to psychiatry, psychology, neurology, mental health, psychiatrist, psychologist, or neurologist using a search engine that has access to 100% of publicly available tweets. Our study timeframe spans from X’s emergence in 2007 to the commencement of our study in 2023, facilitating a comprehensive exploration and analysis of public opinion over an extended period. Our inclusion criteria for tweets in our study were as follows: 1) Tweets including the aforementioned keywords; 2) Public accessibility; 3) Written in either Spanish or English; 4) Publication within the temporal span ranging from 2007 to 2023. We also collected data complementary to the tweets, such as tweet publication date, description of the user’s profile, and the number of retweets and likes generated by each tweet, as an indicator of generated interest of the tweet’s content.

### Natural language processing and topic modelling application

This study employed an unsupervised learning approach using Linear Discriminant Analysis (LDA). Prior to inputting the data into the LDA model, a comprehensive data preprocessing procedure was executed. This preprocessing encompassed language classification, where Spanish tweets were segregated from others, and subsequent translation of the remaining tweets into English via the Google Translate application. Following this, data cleansing activities included the removal of stop words, duplicated words, and extraneous textual elements (e.g., numerical values, abbreviations, hashtags, emojis, and non-standard characters). To determine the optimal number of topics in the topic modelling, a cluster validity index (CVI) (20) was employed, with a specific focus on the silhouette coefficient (ranging between -1 and 1), where higher values indicate superior clustering performance. The silhouette coefficient was chosen due to its capacity to assess both intra-cluster and inter-cluster distances (21). Subsequently, LDA was executed across seven different terms for both the Spanish and English datasets, resulting in a total of 14 topic models. Lastly, sentiment analysis was performed utilizing models from Hugging Face’s machine learning platform, namely “Emotion English DistilRoBERTa-base” (22) for the English dataset and “Beto emotion analysis” (23, 24) for the Spanish dataset, based on the BETO Base model. These models facilitated the categorization of tweets within each topic into Ekman’s six fundamental emotions: anger, disgust, fear, joy, sadness, surprise but adding the neutral emotion (25).

### Ethical considerations

This study was initially reviewed by the University of Alcala Research Ethics Committee and was determined to be a study that did not involve patients. This study was compliant with the research ethics principles of the Declaration of Helsinki (seventh revision, 2013).

## Results

### Mental health takes center stage in both languages, followed by psychiatrists in English tweets, and psychologists in the case of Spanish tweets

A total of 403,767 tweets containing the keywords “psychology,” “psychiatry,” “neurology,” “neurologist,” “psychiatrist,” “psychologist,” and “mental health” were subject to analysis. Among these, 38% (155,217 tweets) were in English, while 62% (248,550 tweets) were in Spanish. All tweets were included in the analysis as they met the inclusion criteria. Notably, the English corpus featured 53,101 tweets (34%) related to “mental health,” surpassing other terms in frequency. “Psychiatrist” garnered 36,400 tweets (23%), and “psychologist” had 29,548 mentions (19%). Only 11% of the tweets referred to “psychiatry” and 4% to “psychology”. In the Spanish dataset, the term “mental health” was present in over 105,504 tweets (42%). “Psychologist” ranked as the second most frequently mentioned term with 66,837 tweets (27%), followed by “psychology” with 36,954 mentions (15%), and “psychiatrist” with 24,009 instances (9.7%). Tweets about “psychiatry” in Spanish comprised only 3,1% of the tweets.

**Figure 1:**
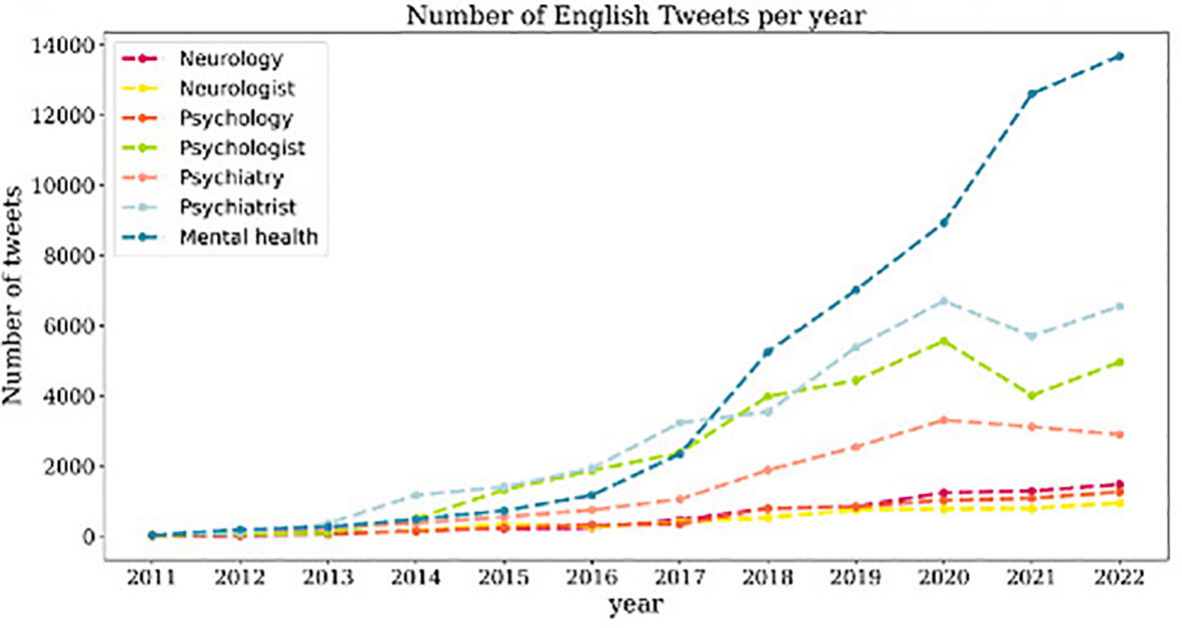
Evolution of number of tweets in English per year. Evolution of English tweets pertaining to the studied terms, each represented by a distinct color, from 2011 to 2022.

**Figure 2:**
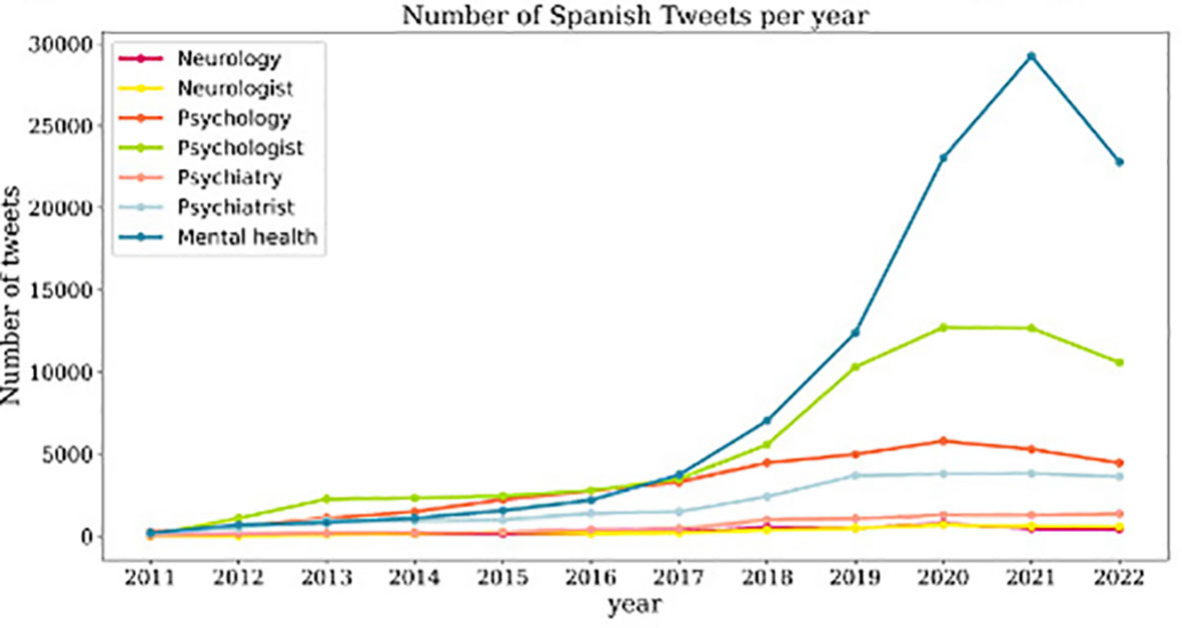
Evolution of number of tweets in Spanish per year. Evolution of the volume of Spanish tweets about the terms studied, with each term represented by a distinct color, from 2011 to 2022.

The general trend for most of the studied terms is a consistent number of tweets from 2011 to 2016, followed by a gradual increase from 2016 to 2022. In the case of English tweets, “mental health,” “psychologist,” and “psychiatrist” exhibit the most pronounced increase in the number of tweets, especially from 2019 onward (**Figure 1**). Concerning Spanish tweets, it is noteworthy that only tweets related to “mental health” and “psychologist” show a progressive increase, while the number of tweets related to other terms remains relatively stable over the years. In 2021, there was a significant surge in the number of tweets about “mental health” which subsequently begins to gradually decrease (**Figure 2**).

When comparing English tweets discussing healthcare professionals, the temporal trend indicates a greater number of tweets mentioning “psychiatrist” when compared to those discussing “psychologist,” although the difference in tweet count is not significantly substantial (**Figure 1**). However, when examining Spanish tweets, the term “psychologist” shows a more pronounced increase in tweet volume over time (**Figure 2**). Moreover, the terms “psychologist” and “psychiatrist” received more attention in tweets than the term “neurologist” throughout the years in both English and Spanish tweets.

In relation to tweets discussing health care fields, we observe distinct trends when comparing English and Spanish tweets. Concerning English tweets, there is a higher number of tweets and a more pronounced increase over time related to the term “psychiatry” compared to tweets containing the term “psychology” and “neurology”, which exhibit significant stability over time (**Figure 1**). In Spanish, while the number of tweets referring to the term “psychology” remains relatively constant throughout the years, they outnumber those mentioning the term “psychiatry” (**Figure 2**).

### Topic modelling

**Figure 3:**
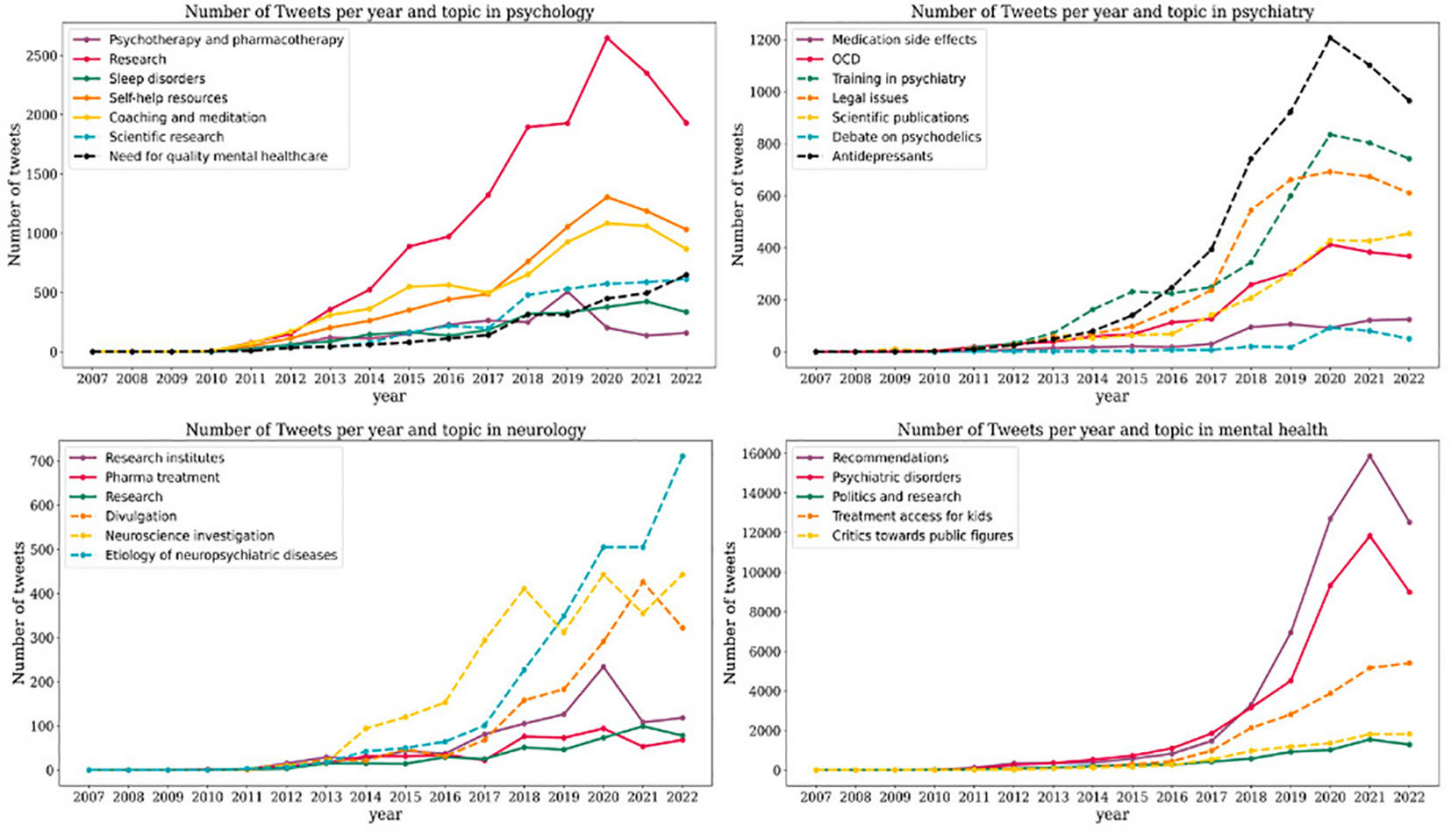
Evolution of the volume of tweets for each topic associated to mental health disciplines (psychology, psychiatry, and neurology) and mental health in general. In the top-left panel, the temporal evolution of the number of tweets generated by topics most frequently associated with Psychology is shown. In the top-right panel, the temporal evolution of the number of tweets generated by topics most frequently associated with Psychiatry is depicted. In the bottom-left panel, the temporal evolution of the number of tweets generated by topics most frequently associated with Neurology is presented. In the bottom-right panel, the number of tweets generated by topics most frequently associated with mental health is displayed. In all four panels, tweets in English are represented by dashed lines, and tweets in Spanish are represented by solid lines.

In Spanish, the most common themes associated with the term “mental health” were recommendations for promoting mental health, psychiatric disorders, and politics and research on mental health. In English, the most frequent topic was treatment access for children with mental disorders, followed by the topic critics towards public and political figures (**Figure 3**). In Spanish, the most common theme when analyzing the term “psychology” is research in psychology. The next two most common topics with a similar number of tweets refer to self-help resources for mental disorders and coaching and meditation. Less-discussed topics include sleep disorders and psychotherapy and pharmacotherapy as treatments for mental disorders. In English, the two most spoken topics spoken with the same frequency are scientific research about mental disorders and the need for good practices in psychiatry and psychology (**Figure 3**).

Regarding the term “psychiatry”, most tweets in Spanish revolve around “side effects of medication”. The fifth most discussed topic is obsessive-compulsive disorder. When analyzing English tweets about “psychiatry”, most of them refer to antidepressants, followed by conversations around training in psychiatry, legal issues and scientific publications (**Figure 3**). Finally, concerning neurology, the most frequent topic in Spanish is research followed by neurology research institutes and pharmacological treatments. In English, we found two prominent topics: neurology and neuroscience research and etiology of neuropsychiatric diseases (**Figure 3**).

**Figure 4:**
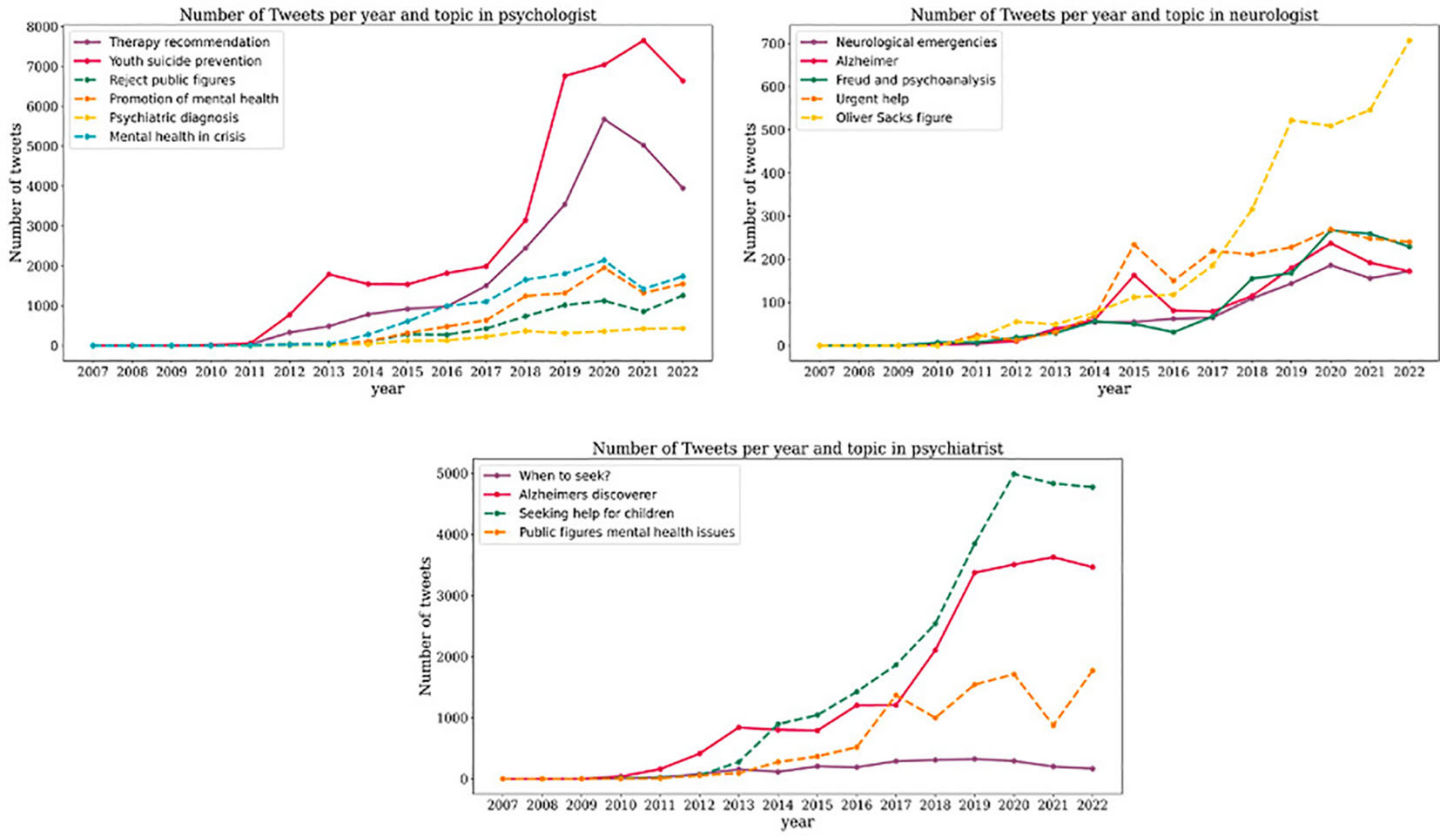
Evolution of the volume of tweets for each topic associated to mental health professionals (psychologist, psychiatrist, neurologist). In the upper-left panel, we illustrate the temporal evolution of the number of tweets generated by topics most frequently associated with Psychologist. In the upper-right panel, we showcase the chronological trend of tweets stemming from topics most frequently associated with Neurologist. In the lower panel, the temporal evolution of tweets emerging from topics most frequently associated with Psychiatrist is depicted. Across all three panels, dashed lines represent tweets in English, while solid lines represent tweets in Spanish.

Concerning mental health professionals, various themes emerge based on the professionals and language. Regarding the term “psychologist”, although the most discussed topic in Spanish is therapy recommendation with over 40,000 tweets, more than 20,000 tweets address suicide prevention in young individuals. In English, several topics arise regarding “psychologist” with the most prominent being mental health in times of crisis followed by promoting mental health in students. On the other hand, the least discussed topic relates to psychiatric diagnosis (**Figure 4**).

Regarding the term “psychiatrist”, the majority of tweets in Spanish revolve around the theme scientist who discovered Alzheimer while only a minority discuss when to consult a psychiatrist. In English, more than 25,000 tweets address seeking help for children with mental disorders, and although less discussed, the remaining tweets pertain to conversations around detrimental mental health in public figures. Lastly, in Spanish tweets about neurologist, two main topics stand out, namely Freud and psychoanalysis and Alzheimer’s disease. The least discussed topic is neurological emergencies. English tweets about “neurologist” primarily focus on Oliver Sacks, a public figure in mental health while the rest are about urgent help from a neurologist (**Figure 4**).

### Temporal evolution of topics

In Spanish mental health-related tweets, recommendations for promoting mental health and discussions about psychiatric illnesses have been the most discussed topics, with a notable increase starting in 2018 and the highest peak occurring in 2021. In English tweets, access to treatment for children with mental disorders shows varying prominence over the years, with a significant increase from 2016, reaching its peak in 2022 (**Figure 3**).

Regarding healthcare professions, variations exist depending on the profession and language. In Spanish tweets discussing “psychology” topics such as psychology research, self-help resources for psychiatric disorders, and coaching and meditation have shown an increasing trend, notably in 2020. In English tweets, both of the topics found exhibit a growing number of tweets. While tweets related to scientific research about mental disorders have been more frequent over the years compared to those about the need for adequate practices in psychiatry and psychology, 2022 saw a decrease in the former and a continued increase in the latter (**Figure 3**). Analyzing Spanish tweets about “psychiatry” over time, there has been an increase in discussions related to obsessive-compulsive disorder, particularly in 2020. The number of tweets about medication side effects has remained relatively low and stable over time, with a slight increase in 2021 and 2022. In English tweets, discussions about antidepressants, training in psychiatry, legal issues, and scientific publications have exhibited an increasing trend, with a peak in tweets about antidepressants in 2019, followed by a gradual decline. Topics related to training in psychiatry, legal issues, and scientific publications have significantly increased since 2017, with a higher concentration of tweets between 2019 and 2022 (**Figure 3**). Regarding English tweets about “neurology” the most common topics over time include neurology and neuroscience research and the etiology of neuropsychiatric diseases. Tweets discussing the etiology of neuropsychiatric diseases became the most discussed topic between 2020 and 2022. In Spanish, tweets about Neurology research institutes reached their highest number between 2019 and 2020 and subsequently decreased. In 2018, there was an increase in tweets discussing pharmacological treatments, but it significantly declined after 2020 (**Figure 3**).

When analyzing tweets in Spanish about “psychologists”, conversations about recommendation to go to therapy and suicide prevention in young people increased over the years. Both topics reached their highest peak of tweets between 2020 and 2021. In English tweets, the tendency is to an increment of tweets in all the topics through the years. Conversations around promoting mental health in students and mental health in time of crisis become especially relevant in 2020 and 2022 (**Figure 4**). In tweets including the term “psychiatrist” there was a significant increase in the number of tweets in English about seeking help for children with mental disorders between 2019-2022. Conversations around detrimental mental health in public figures followed a trend where there was an increase in one year followed by a decrease in the next (**Figure 4**). In Spanish tweets about “neurologist”, the trend is to an increase in all the topics. As for English tweets, conversations around Oliver Sacks public figure in mental health increased over the years and became substantially relevant in 2022 (**Figure 4**).

### Emotions associated to each topic

**Figure 5:**
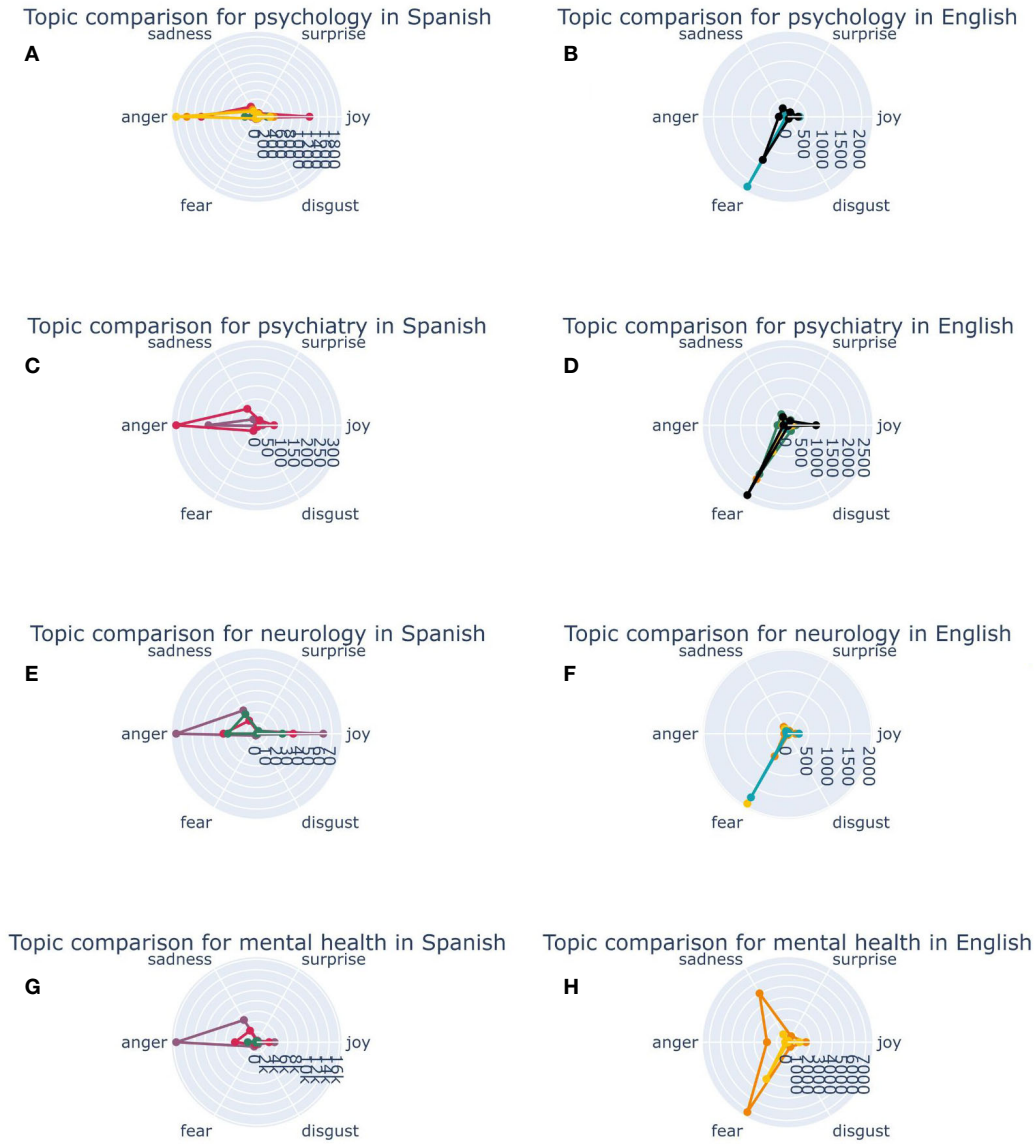
Emotions associated to each topic regarding mental health disciplines. In panel **(A)**, Spanish topics related to Psychology alongside their respective associated emotions are shown. In panel **(B)**, English topics associated with Psychology and the emotions they elicit are represented. In panel **(C)**, topics related to Psychiatry in Spanish and their corresponding associated emotions are illustrated. In panel **(D)**, English topics emerging from Psychiatry and their respective associated emotions are visible. In panel **(E)**, topics in Spanish related to Neurology and the emotions they evoked are shown. In panel **(F)**, English topics referring to Neurology and their corresponding associated emotions are represented. In panel **(G)**, topics about mental health in Spanish and the emotions they evoked are showcased. In panel **(H)**, English topics about mental health and their associated emotions are displayed.

Regarding the term “mental health”, most of the tweets in Spanish referred to recommendations to promote mental health associated to anger, while only a few were associated to sadness (**Figure 5**). In English, tweets referred to treatment access for children with mental disorders are predominantly linked to fear and sadness (**Figure 5**). In regard to “psychology”, conversations in Spanish about research are linked to joy and anger, while conversations about self-help resources for mental illnesses are associated to anger mostly. When examining tweets in English about “psychology”, conversations about Scientific research about mental disorders and the need for adequate practices in psychiatry and psychology were predominantly associated with fear. In tweets in English that include the term “psychiatry” it is relevant that conversations around antidepressants generated fear. In Spanish tweets about “psychiatry”, the predominant sentiment when tweeting about side effects of medication and obsessive-compulsive disorder is anger. Tweets about “neurology” in English, on the other hand, show that conversations about neurology research and the etiology of neuropsychiatric diseases generate fear. Tweets in Spanish that discussed about “neurology research institutes” mostly caused feelings of anger (**Figure 5**).

**Figure 6:**
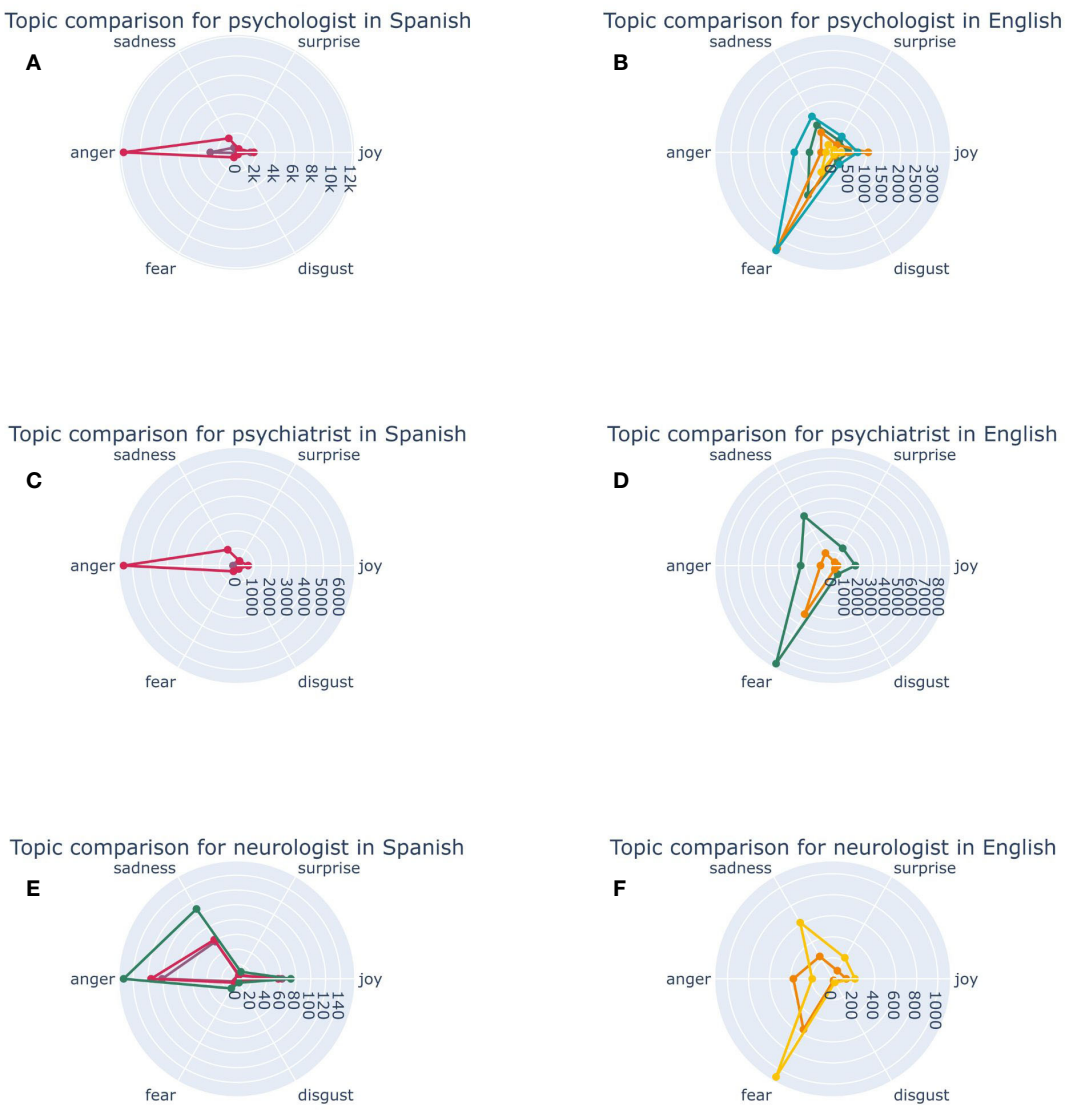
Emotions associated to each topic regarding mental health professionals. In panel **(A)**, Spanish topics related to Psychologist alongside their respective associated emotions are represented. In panel **(B)**, English topics associated with Psychologist and the emotions they evoked are displayed. In panel **(C)**, topics derived from Spanish tweets concerning Psychiatrist along with the corresponding associated emotions are presented. In panel **(D)**, topics related to Psychiatrist in English and the emotions they elicit are illustrated. In panel **(E, F)**, topics stemming from Neurologist in both Spanish and English, respectively, and the emotions linked to each topic are showcased.

Tweets in English about “psychologist” that discussed promoting mental health and mental health in time of crisis are all linked to fear (**Figure 6**). On the other hand, tweets in Spanish about suicide prevention in young people were all associated with anger. When exploring tweets about “psychiatrist” in English, conversations around seeking help for children with mental disorders caused primarily fear, but also sadness. In Spanish tweets, conversations around scientist that discovered Alzheimer were linked to anger. Tweets about “neurologist” in Spanish that discuss urgent help by neurologist were associated to sadness and fear. In Spanish, tweets about neurology emergencies, Alzheimer and Freud and psychoanalysis generated anger primarily, but also sadness and joy (**Figure 6**).

## Discussion

In our study, the most frequently mentioned term in tweets, both in Spanish and English, is mental health, with an observed increase in the number of tweets mentioning this term over the 15-year study period. However, some differences emerged based on the language of the tweets. In English, the most discussed terms after mental health were psychiatrist and psychologist, while in Spanish, psychologist and psychology ranked second and third in frequency. Additionally, we observed a progressive increase in the number of tweets about these mentioned terms over time, both in Spanish and English, especially starting in 2019.

Various studies using similar methodologies to ours have demonstrated a significant increase in the volume of tweets about mental health during the COVID-19 pandemic, reflecting a heightened concern for mental health among the population (26, 27). A recent meta-analysis also revealed a growing positive attitude toward psychiatry and psychiatric treatment over the past two decades. Public perception increasingly considers psychiatrists as valuable for the treatment of depression and schizophrenia (28). The pandemic caused by COVID-19 may have stimulated discourse on mental health, yet this upward trend was already positioned earlier in 2019, indicating that this topic was already of interest to users. For instance, the expansion of online mental health resources in recent years, including informational websites, support groups, and therapy platforms, may have encouraged greater awareness and conversations about mental health issues among X users. Moreover, influential figures such as celebrities and social media influencers have utilized their platforms to share personal experiences and advocate for mental health awareness. Their influence could have also contributed to the pre-existing rise in mental health-related discussions on X before the COVID-19 pandemic.

It is noteworthy that Spanish tweets addressing mental health sharply declined after 2021, possibly due to user fatigue towards discussions about mental health. A recent study linked active content engagement to pandemic fatigue, explained by information overload and desensitization (29). Applying this insight to our findings, it is plausible that the saturation of mental health information in previous years may have desensitized users, leading to the sudden drop in mental health-related tweets after 2021. Societal attention may have shifted away from the pandemic and mental health issues to other concerns and events, resulting in fewer tweets about mental health on X. An alternative explanation could be that algorithm or feature adjustments within X might have impacted the visibility and prevalence of mental health related discussions, potentially contributing to the decline in tweets about mental health after 2021.

On the other hand, although our study reveals differences in the predominance of tweets about psychiatrist and psychologist, various studies have shown a preference for psychologists over psychiatrists among the public (30–34). Our study showed that the term psychiatry was predominantly associated with the topics ‘antidepressants’ and ‘medication side effects,’ which were both linked to fear and anger. It is possible that users not only associate psychiatry with medication and its side effects but also with the medical professionals who prescribe them. This could explain why people prefer psychologists over psychiatrists, which unlike psychologists, can prescribe these drugs that still evoke fear and anger among the general population.

It is worth noting that, tweets related to the terms neurology and neurologist remained relatively stable over the 15 years covered in our study, displaying a lower frequency of tweets compared to those concerning mental health professionals and professions. The latter showed a trend of increasing over time. Various reasons may explain this increase in conversations about mental health professionals and professions, especially in the last two years. Firstly, the COVID-19 pandemic led to a rise in the prevalence of mental health conditions such as anxiety and depression, along with increased stress, feelings of loneliness, and a decline in emotional and mental well-being (5–10). Personal experiences or those of close acquaintances with such symptoms or psychiatric illnesses, as well as an increased awareness of the impact of these conditions on the quality of life, may have fueled discussions, not only about these conditions but also about psychologists and psychiatrists, who are responsible for diagnosis, prevention, and treatment. Additionally, various media outlets on social networks have also promoted discussions around psychotherapy, extending the reach of these topics. A recent study observed an increase over the years in the number of tweets published by leading US media outlets on various psychotherapies over the last 11 years (35). This highlights how media coverage plays a significant role in shaping public discourse, contributing to increased awareness and receptivity toward treatments for mental illnesses, such as psychotherapy and pharmacotherapy.

Interesting results emerged from topic modelling depending on the analyzed term and the language of the tweets. Users discussing mental health in Spanish were primarily interested in recommendations to promote mental health and psychiatric disorders, while English-speaking users showed more interest in discussing treatment access for children with mental disorders. Conversations on these topics increased substantially over the years, particularly in 2021-2022. Social media platforms are a powerful tool for combating the stigma associated with mental illnesses and promoting both discussions on mental health and help-seeking behaviors in the face of emotional difficulties (36–38). Adolescents, in particular, often encounter resistance when seeking help for emotional distress due to the stigma associated with these conditions or a lack of trust in the healthcare system (39). Social media has become an anonymous space where young people seek support for emotional distress, constituting a community where they feel safer sharing their experiences, offering advice, and finding solace in the personal stories of celebrities or public figures about their emotional struggles (40). Sharing recommendations on how to address certain emotional difficulties on X may have become a useful tool for many, especially in the midst of the healthcare system strain partly due to the overwhelming demand on medical facilities during the pandemic and the barriers to accessing mental health treatment stemming from the economic repercussions of recessions experienced during this period. Additionally, social media features mass media campaigns, which may have played a significant role in promoting mental health recommendations, as they have been shown to have the capacity to produce positive changes or prevent negative changes in health-related behaviors (41).

Moreover, in recent years, a notable theme for X users has been the demand for appropriate and effective psychological and psychiatric practices, which has coincided with the proliferation of self-help resources. While these resources cannot replace individualized and continuous psychological or psychiatric treatment, they are considered a valuable support for mental health (42). Individuals affected by crises use X as a means of emotional release, reflecting collective concerns through expressions of anxiety or fear (43, 44). In fact, in a recent study, one of the dominant themes was related to the right to access quality healthcare (18). Tweets in our study may mirror these collective concerns and a growing public demand for enhanced psychological and psychiatric practices, particularly amid recent rises in mental illness prevalence, potentially indicating heightened public awareness of the necessity for effective treatment.

Regarding psychiatry, conversations in Spanish primarily revolved around the side effects of medication, a topic that, although stable in the initial years of the study, increased significantly in 2021 and 2022. English-speaking users showed particular interest in antidepressants, experiencing significant growth in 2019-2020. It is worth noting that discussions surrounding antidepressants or side effects of medication became more prominent after 2019, coinciding with the increased use of psychotropic medications worldwide. Several studies demonstrate that the prescription and use of psychotropic medication increased during and after the COVID-19 pandemic, affecting both adolescents and young adults and correlating with an increase in psychiatric disorders (44, 45). One study showed a significant increase in the prevalence of antidepressants and antipsychotics in the last months of 2020 (46). The increased use of these drugs, particularly antidepressants, has been observed across diverse age groups in both men and women (46, 47). The surge in antidepressant use likely prompted user curiosity regarding their mechanisms, side effects, and efficacy. While attitudes toward antidepressant usage vary, multiple studies underscore a prevalent discourse characterizing them as harmful, addictive, and inefficacious (48–50). Medication-related stigma poses a significant obstacle to treatment adherence, potentially leading users to irregular or insufficient dosage, thereby increasing the likelihood of side effects and diminishing effectiveness (51, 52). Communicating and promoting accurate information through social media about mental illnesses and their pharmacological treatment can dispel myths and prejudices (53, 54). Indeed, one study demonstrated that educating participants about depression and its pharmacological treatment, as well as showing them videos of patients who have benefited from antidepressants, generated a more positive attitude toward people with depression and their treatment (52).

Finally, sentiment analysis revealed that the majority of the analyzed terms were associated with negative emotions, such as fear, anger, or sadness. The stigma surrounding mental illnesses and those who suffer from them is still evident in common assumptions that these conditions and individuals are unpredictable and dangerous (54, 55). On X, although it may seem that the discourse about various aspects of mental health, such as seeking help, promoting mental health, and medication use, has increased, the negative emotions associated with these topics persist. However, it is encouraging that despite the negative emotions that discussing these issues can provoke, the trend has been toward an increase in these conversations, which are essential for raising awareness about mental illnesses and combating the stigma associated with mental health. Although one might assume that negative sentiments could be associated with a stigmatizing attitude, it appears that predominant topics found in our study reflect a trend toward promoting mental health.

### Limitations

One limitation of our study pertains to the non-representativeness of tweets in reflecting broader societal attitudes toward mental health or mental health professionals and disciplines. This is because conversations on the studied constructs may not capture the perspectives of individuals who do not use X or do not actively engage in mental health discussions on the platform. Additionally, X’s character limit constraints may oversimplify or lack nuance in discussing complex topics like mental health professionals and disciplines, potentially leading to a superficial understanding of user sentiments. Furthermore, X conversations lack the contextual depth provided by face-to-face interactions or qualitative interviews, hindering the comprehension of underlying meanings behind user statements about mental health professionals and disciplines. Future research could enhance these findings by incorporating data from other social media platforms, and employing alternative data collection methods, such as in-depth interviews.

## Conclusions

Our study provides valuable insights into the evolving landscape of mental health discussions on X over a 15-year period. Mental health emerges as the most frequently mentioned term in both Spanish and English tweets, reflecting a growing societal awareness of the importance of mental well-being. Even though our results reveal variations in user interests and a prevalence of negative sentiments, it underscores the importance of ongoing efforts to destigmatize mental health, increase access to resources, and foster a more compassionate and informed society.

Social media platforms offer vital avenues for advocacy and education, and the upward trajectory of these conversations indicates a promising future for mental health awareness. By addressing societal concerns, adapting to user interests, and harnessing the potential of online discourse, we can collectively work towards a society that places mental health at the forefront of our collective well-being, reducing stigma and increasing support. Finally, the persistence of negative sentiments in mental health discussions highlights the continued importance of challenging stigma and fostering empathy. As society progresses and digital platforms continue to play a pivotal role in shaping public discourse, our study underscores the need for ongoing dialogue and advocacy, emphasizing the critical role of both professionals and the broader community in advancing mental health awareness and support.

## Data availability statement

The raw data supporting the conclusions of this article will be made available by the authors, without undue reservation.

## Author contributions

JD-E: Conceptualization, Investigation, Writing – original draft, Writing – review & editing, Data curation. AV: Supervision, Writing – review & editing. MM: Data curation, Formal analysis, Methodology, Writing – review & editing. FL-A: Data curation, Formal analysis, Methodology, Writing – review & editing. LG-R: Supervision, Validation, Writing – review & editing. EdC: Supervision, Validation, Writing – review & editing. RR-J: Supervision, Validation, Writing – review & editing. MdC: Supervision, Validation, Writing – review & editing. MO: Supervision, Validation, Writing – review & editing. MA-M: Conceptualization, Supervision, Validation, Writing – review & editing. MAA-M: Writing – review & editing.
